# Effects of short-term inpatient treatment on sensitivity to a size contrast illusion in first-episode psychosis and multiple-episode schizophrenia

**DOI:** 10.3389/fpsyg.2013.00466

**Published:** 2013-07-24

**Authors:** Steven M. Silverstein, Brian P. Keane, Yushi Wang, Deepthi Mikkilineni, Danielle Paterno, Thomas V. Papathomas, Keith Feigenson

**Affiliations:** ^1^Rutgers University Behavioral Health Care, and Department of Psychiatry, Robert Wood Johnson Medical School, Rutgers Biomedical and Health SciencesPiscataway, NJ, USA; ^2^Center for Cognitive Science, Rutgers UniversityNew Brunswick, NJ, USA

**Keywords:** schizophrenia, vision, perception, cognition, state marker, biomarker, context, disorganization symptoms

## Abstract

**Introduction:** In the Ebbinghaus illusion, a shape appears larger than its actual size when surrounded by small shapes and smaller than its actual size when surrounded by large shapes. Resistance to this visual illusion has been previously reported in schizophrenia, and linked to disorganized symptoms and poorer prognosis in cross-sectional studies. It is unclear, however, when in the course of illness this resistance first emerges or how it varies longitudinally with illness phase.

**Method:** We addressed these issues by having first-episode psychosis patients, multiple-episode schizophrenia patients and healthy controls complete a psychophysical task at two different time points, corresponding to hospital admission and discharge for patients. The task required judging the relative size of two circular targets centered on either side of the screen. Targets were presented without context (baseline), or were surrounded by shapes that made the size judgment harder or easier (misleading and helpful contexts, respectively). Context sensitivity was operationalized as the amount of improvement relative to baseline in the helpful condition minus the amount of decrement relative to baseline in the misleading condition.

**Results:** At hospital admission, context sensitivity was lower in the multiple-episode group than in the other groups, and was marginally less in the first episode than in the control group. In addition, schizophrenia patients were significantly more and less accurate than the other groups in the misleading and helpful conditions, respectively. At discharge, all groups exhibited similar context sensitivity. In general, poorer context sensitivity was related to higher levels of disorganized symptoms, and lower level of depression, excitement, and positive symptoms.

**Discussion:** Resistance to the Ebbinghaus illusion, as a characteristic of the acute phase of illness in schizophrenia, increases in magnitude after the first episode of psychosis. This suggests that visual context processing is a state-marker in schizophrenia and a biomarker of relapse and recovery.

## Introduction

Recent years have seen a renewed interest in visual perception in schizophrenia. Reasons for this include: (1) vision is arguably the best understood domain of mental functioning (Palmer, 1999); (2) reliable and valid measures from the field of vision science are available to assist with answering specific questions about brain and cognitive functioning in schizophrenia (Butler et al., 2008, 2012); (3) studies have consistently demonstrated specific perceptual differences between people with schizophrenia and matched controls (Butler et al., 2008; Chen, 2011; Green et al., 2011; Silverstein and Keane, 2011a), and this can be done independently of a generalized deficit in many cases (Place and Gilmore, 1980; Knight, 1984; Knight and Silverstein, 2001; Dakin et al., 2005; Koethe et al., 2009; Yoon et al., 2009; Tibber et al., 2013); (4) patterns of abnormal regional activation, connectivity/circuitry, and/or neurotransmitter activity have been associated with visual impairments in schizophrenia, and these are consistent with what is known about normal vision from the neurobiology literature (Spencer et al., 2004; Silverstein et al., 2009; Sehatpour et al., 2010; Uhlhaas and Singer, 2010; Yoon et al., 2010; Butler et al., 2013; Plomp et al., 2013); (5) theoretical models and empirical data link visual impairments with aspects of behavioral and cognitive functioning, in some cases suggesting that perceptual impairments are low-level manifestations of widespread canonical computations that are impaired in the disorder (Phillips and Silverstein, 2003, 2013; Silverstein and Keane, 2011a,b); (6) visual impairments are related to problems in daily functioning in schizophrenia (Green et al., 2012); and (7) some visual abnormalities in schizophrenia are related to clinical state (Silverstein et al., 1996; Uhlhaas et al., 2005; Keane et al., 2013), suggesting they may be biomarkers of relapse, recovery, or treatment response, whereas other abnormalities are stable over time and can be found in unaffected relatives, suggesting they may be genetic or endophenotype markers (Yeap et al., 2006).

**Figure 1:**
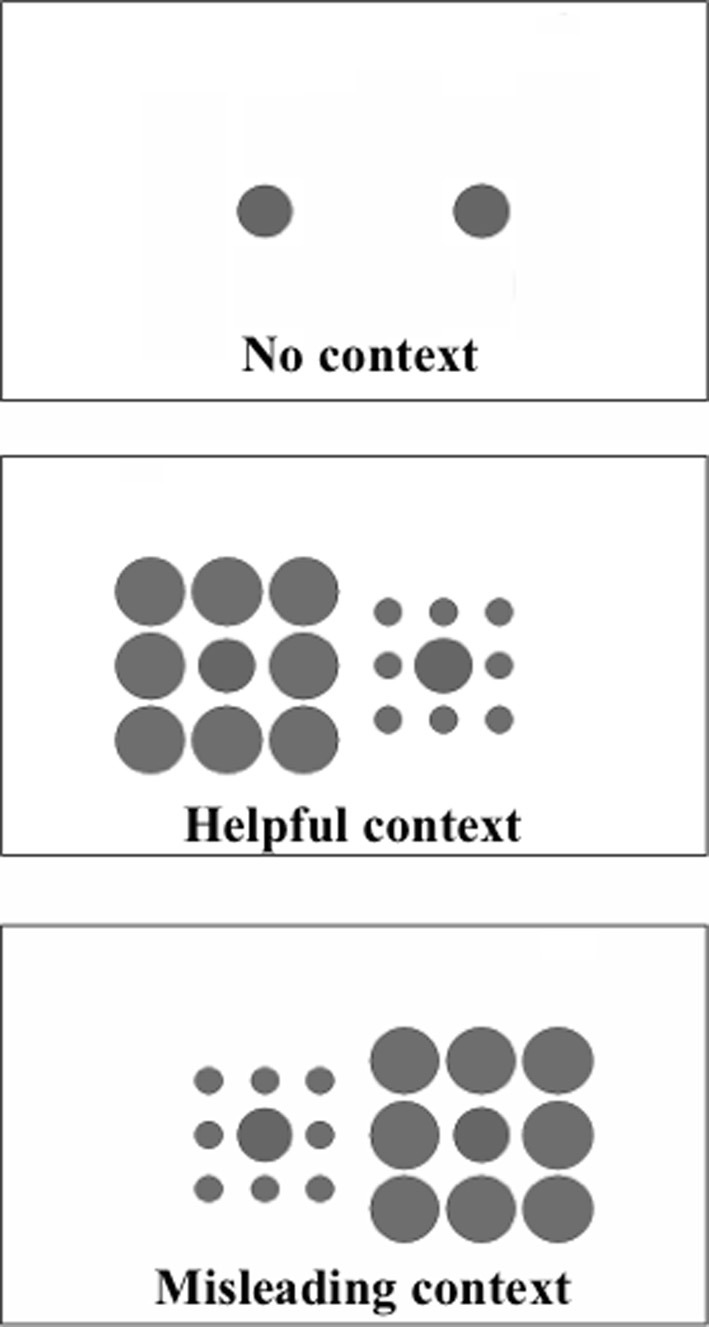
**Examples of the stimuli shown in each of the context conditions (the text was not present in the displays, only the circles)**. On each trial participants were presented with a display from one of the conditions, and were asked to indicate, via a keypress, which circle (or, in the misleading and helpful conditions, which inner circle) was largest, the one on the left or the one on the right. In each case shown here the inner circle on the right is 2% larger than that on the left. Figure reprinted, with permission by John Wiley and Sons, from Doherty et al. (2010).

**Figure 2:**
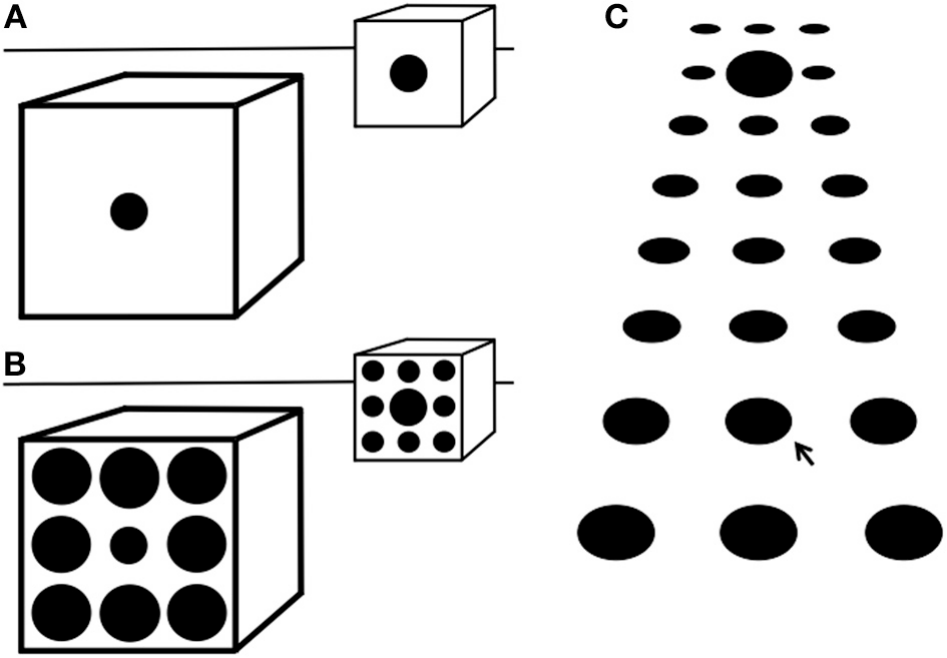
**(A)** Most people see the further circle as being larger than the nearer one, though they are equal. They would also judge the ‘real’ size of the further circle within the pictured space to be much larger than the nearer circle. This shows that pictorial cues to depth and size influence perception of the markings on the picture surface. **(B)** Adding surrounds, as in the Ebbinghaus illusion, increases the perceived size difference between the two circles. This suggests that surround size adds to the other pictorial depth cues. **(C)** In texture gradients the mean size and separation of elements decreases with depth. The size of the elements on the picture surface is seen as decreasing with depth, but their ‘real’ size within the pictured space would be judged to be approximately constant. The large element in the center of the second row from the top may be seen as being larger than that arrowed below, but they are equal. Its ‘real’ size within the pictured space would be judged to be much larger. The bottom and top three rows are versions of the Ebbinghaus illusion. Therefore, this suggests that the illusion may in part be due to the visual system learning to use such pictorial cues. Figure reprinted, with permission by John Wiley and Sons, from Doherty et al. (2010).

The primary goal of this study was to examine whether scores on an index of visual context processing covary with clinical state over the course of short-term inpatient treatment. To do this, patients were tested at admission and discharge/transfer from an acute care inpatient unit. The visual task involved a variant of the Ebbinghaus illusion in which a circle appears larger than its actual size when surrounded by smaller circles, and smaller than its actual size when surrounded by larger circles (see Figures 1, 2). On each experimental trial, subjects were shown two target circles—one on the left of the screen and one on the right—and the task was to decide which was larger. On half of the trials, the targets were surrounded by larger or smaller circles that would make giving a correct response easier (helpful condition) or harder (misleading condition; see Figure 1). As discussed further below, we chose this illusion because it has been established over decades of research, because it is experienced to a lesser extent among schizophrenia patients relative to healthy and psychiatric controls (Uhlhaas et al., 2006a,b; Tibber et al., 2013), and because reduced illusions have been linked to a more acutely ill clinical state and to more disorganized symptoms in cross-sectional studies (Uhlhaas et al., 2006a,b; Horton and Silverstein, 2011). Another advantage to this task is that it can side-step generalized deficit confounds, in which low accuracy can be attributed to reduced motivation or attention (Knight and Silverstein, 2001; Silverstein, 2008). In the Ebbinghaus task, patients are expected to perform *better* than healthy controls in the misleading context condition, worse than controls in the helpful context condition, and about the same in the no-context condition.

The Ebbinghaus illusion was discovered by German psychologist Hermann Ebbinghaus (b. 1850, d. 1909), was popularized by Edward Titchener's 1902 psychology textbook (Titchener, 1902), and has been the subject of numerous experiments since the 1970s (e.g., Massaro and Anderson, 1971; Girgus et al., 1972; Weintraub and Schneck, 1986; Coren and Enns, 1993; Rose and Bressan, 2002; Doherty et al., 2010; Schwarzkopf and Rees, 2013). The illusion depends on basic stimulus parameters such as the relative sizes of target and surrounds, distances between targets and surrounds, and differences in form between targets and surrounds (Massaro and Anderson, 1971; Choplin and Medin, 1999). It can also be affected by the conceptual similarity between target objects and their surrounds (Coren and Enns, 1993) or by affective cues (Van Ulzen et al., 2008). However, in the absence of such high-level manipulations, the illusion is thought to primarily reflect: (1) perceptual organization, since the illusion requires integration of targets and surrounds (Kovacs, 2000); and (2) size constancy, which involves “top-down” effects of prior knowledge of depth cues on the representation of sensory input (Phillips et al., 2004; Doherty et al., 2008, 2010; Caparos et al., 2012). Regarding the latter, when the surrounding circles are larger than the center target circle, this creates the implicit assumption that the stimulus set is relatively close to the observer, and the center object is therefore perceived as smaller than its actual size. In contrast, smaller surrounds lead to the implicit assumption that the stimulus set is relatively far from the observer, and the center object is then perceived as larger than its actual size (see Figure 2) (Doherty et al., 2010). These effects are consistent with the tendency, in adults, to overestimate the size of distant objects and to underestimate the size of near objects (Kavsek and Granrud, 2012). The Ebbinghaus illusion can also be considered a form of surround suppression, in the sense that perception of a central target is modulated by surrounding context in a direction opposite to characteristics of the surround (Tibber et al., 2013).

A second goal of this study was to compare the performance of people with a first episode of psychosis to that of healthy controls and people with an established diagnosis of schizophrenia. To date, there have been no studies of size contrast illusions—or any other type of surround suppression—in first-episode psychosis. It is therefore unclear whether reduced illusion effects, and the abnormalities in neural mechanisms that subserve these reductions, are an aspect of psychosis in general, schizophrenia in general, or psychosis and/or illness progression in schizophrenia. However, evidence suggests that perceptual organization impairments are associated with illness chronicity and progression. For example, reduced perceptual organization has been observed among patients requiring long-term hospitalization compared to patients requiring short-stays who can usually function in the community, with the latter group performing normally (Silverstein et al., 1998, 2006b; Uhlhaas et al., 2006b; Silverstein and Keane, 2011a). At the same time, past studies in prodromal or first-episode schizophrenia indicate that perceptual organization is intact at those time points (Parnas et al., 2001; Silverstein et al., 2006a). Because the evidence so far is limited to two studies (both cross-sectional) and because no study has explicitly examined surround suppression (let alone size contrast illusions) in first episode patients, however, it remains an open question as to how persons with first episode psychosis perform relative to our other groups on the type of task we report on here.

## Method

### Subjects

Three subject groups participated: (1) patients hospitalized for their first episode of psychosis (FEP) (*n* = 16, 9 males), and so, for whom, the eventual diagnosis (e.g., mood disorder with psychotic features vs. schizophrenia spectrum disorder) is unknown at this time point; (2) patients in their second or later episode of schizophrenia (SCZ) (*n* = 21, 16 males) recruited from the same short-term inpatient unit as the first episode subjects; and (3) healthy controls (CON) screened to rule out the presence of a psychotic or mood disorder (*n* = 27, 14 males). Demographic characteristics and symptom profiles of each group can be found in Table 1. For the FEP group, research diagnoses (and *n*) on admission were as follows: psychotic disorder NOS (8), schizophrenia (1), schizoaffective disorder (1), delusional disorder (1), and major depression with psychotic features (5). Average length of stay on the inpatient unit for patients was 16.46 days (*SD* = 8.10, median = 15.00). On average, patients were initially tested 5.28 days after hospital admission (*SD* = 4.54, median = 4.00), and then again 13.60 days later (*SD* = 6.64, median = 13.00). To be included in the study, patients had to be between the ages of 18–60, and had to be diagnosed with either schizophrenia, or a first episode of a psychiatric disorder with psychotic symptoms. Exclusion criteria included: (1) any history of TBI or head injury with loss of consciousness greater than 10 min; (2) history of a neurological or developmental disorder; (3) current mood disorder; (4) current substance abuse or dependence disorder (within past 6 months) or positive urine toxicology screen on any day of testing; (5) estimated premorbid (Wechsler) IQ < 70, as determined by the Shipley Institute of Living Scale (Shipley et al., 2009) or evidence of intellectual disability as indicated in the electronic medical record; or (6) ECT within the past 8 weeks. All patients were receiving antipsychotic medication. Exclusion criteria for the CON group included those listed for patients, as well as: (1) any lifetime Axis-I disorder (as assessed by SCID) with the exception of past substance use disorders; (2) psychotropic medication use in the last 6 months; and (3) a first-degree relative(s) with a diagnosis of schizophrenia, schizoaffective disorder, or bipolar disorder (based on subject self report). All subjects had normal or corrected-to-normal visual acuity as assessed via a Snellen chart.

**Table 1:** **Demographic and clinical characteristics of participants**.

**Variable**	**FEP**		**SCZ**		**CON**	
	**Mean**	***SD***	**Mean**	***SD***	**Mean**	***SD***
Age (in years)	26.38	9.37	42.62	12.21	41.87	11.90
Gender (% Male)	56.20		76.20		54.80	
Ethnicity (% Caucasian)	31.20		52.40		25.80	
Personal education (in years)	13.38	3.30	12.19	1.44	14.16	2.37
Father education (in years)	13.86	3.84	12.94	3.89	12.70	4.63
Mother education (in years)	13.00	4.61	12.11	3.89	12.58	4.25
PANSS Positive time 1^a^	10.13	4.53	11.76	4.25		
PANSS Negative time 1^b^	16.53	6.16	17.76	4.23		
PANSS Cognitive time 1^c^	10.67	4.30	13.86	4.89		
PANSS Depression time 1^c^	13.33	4.47	12.48	5.00		
PANSS Excitement time 1^a^	9.73	2.34	8.71	3.26		
PANSS Positive time 2^a^	7.71	3.05	10.29	3.75		
PANSS Negative time 2^b^	10.07	4.60	15.93	4.39		
PANSS Cognitive time 2^c^	8.79	2.26	11.64	3.95		
PANSS Depression time 2^c^	10.79	5.19	12.79	3.89		
PANSS Excitement time 2^a^	7.36	2.13	9.21	3.29		

a * Factor score based on a 4-item scale (each item rated 1–7)*.

b * Factor score based on a 6-item scale (each item rated 1–7)*.

c * Facor score based on a 5-item scale (each item rated 1–7)*.

#### Apparatus

Stimuli were presented on a Samsung 2243BWX LCD monitor with viewable dimensions of 47.5 by 29.8 cm. The viewing distance was 24 inches (60.9 cm). The screen resolution was 1680 × 1050, and therefore, the viewable screen subtended 43° × 27° of visual angle. Spyder 3 Elite software was used to calibrate the monitors across sites at the start of the study and then monthly afterwards. Monitor parameters were a gamma value of 2.2, color temperature (white point) of 6500K, and luminance of 120 cd/m^2^.

#### Ebbinghaus illusion task

Stimuli were presented and responses were recorded and analyzed with a C++ program developed by Phillips et al. (2004). This task has been used in four prior studies of the Ebbinghaus illusion, including one of schizophrenia (Phillips et al., 2004; Doherty et al., 2008, 2010; Horton and Silverstein, 2011). On each trial, the task was to press a key to indicate whether the target on the left or the right half of the screen was larger (see Figure 1). All circles were black and presented on a white background. The stimulus appeared on the screen until the subject responded or after 2 s (whichever happened first). If a response was not recorded within 2 s of stimulus onset, the trial was counted as a guess (0.5 correct). Trials were separated by 200 ms. The targets were centered on either side of the screen and appeared either with or without surrounding circles (see below). The two target circles always differed in actual size, and this difference varied in magnitude across trials. The center circle on one side was always 2.67° of visual angle in diameter, while the center circle on the other side was always 0.05°, 0.16, 0.27, 0.37, or 0.48° larger or smaller. The side on which the larger circle appeared was randomized. This size comparison was presented in 3 conditions. (1) In the *misleading* condition, the target circles were always surrounded by 8 larger circles arranged in a square configuration (i.e., 3 above, one on each side, and 3 below, see Figure 1). Each of the five size differences was shown sixteen times, with the larger central circle always surrounded by larger circles (3.33° in diameter) and the smaller central circle always surrounded by smaller circles (1.33° in diameter). In this condition, size contrast impairs discrimination by biasing the observer to perceive the larger target as smaller and the smaller target as larger (Doherty et al., 2008). (2) In the *helpful* context condition, the 2.61 and 2.72° target circles were presented eight times each, again surrounded by 8 circles around the edges of an imaginary square, with the smaller center circle surrounded by circles 3.33° in diameter and the larger central circle surrounded by circles 1.33° in diameter. In this condition, size contrast increases accuracy. Note that in this condition, if subjects choose the array with larger *surrounds* then they will be wrong on every trial. As in prior studies, only 16 trials were presented in the helpful condition, and these were all at the hardest difficulty level (0.05° size difference between center circles) (Phillips et al., 2004; Doherty et al., 2008). The 96 trials in the context conditions (80 in the misleading and 16 in the helpful conditions) were presented in a different random order for each subject. (3) In addition to these 96 trials, 96 additional trials were presented in a control (*no-context*) condition, also in a different random order for each subject, using the same 80 size comparisons as in the misleading condition, plus 16 additional trials at the smallest size difference. In other words, the no-context trials were exactly the same as the block of trials with context, except that the surrounding circles were invisible. This block of trials was presented either before or after the trials containing context, with the order of context and no context blocks counterbalanced across subjects. In total, the task contained 192 trials, and typically took less than 10 min to complete.

During the course of the study, it was discovered that the location on the screen of the entire stimulus display would be shifted slightly to the left or right, corresponding to the side that contained the larger target circle. This occurred 83% of the time within the context block (only on misleading trials) and occurred the same percentage of time within the no-context blocks. This heretofore unknown feature of the program is not deemed problematic for our analyses because it occurred with an equal incidence in the helpful and no-context trials (used for the facilitation calculation) and also in the misleading and no-context trials (used for the impairment calculation; see below). Therefore, the facilitation and impairment calculations were not biased by any aspect of the display presentation.

### Clinical assessment measures

All patients were interviewed with the Structured Clinical Interview for DSM-IV Diagnosis (SCID), patient version (First et al., 2002b). Information was also obtained from medical records and discussions with staff to confirm the final research diagnosis. The CON group was screened for psychopathology using the non-patient version of the SCID (First et al., 2002a). For patients, symptoms were rated, based on the past week, using the Positive and Negative Syndrome Scale (Kay et al., 1987), which was scored using a 5-factor model (Lindenmayer et al., 1994a,b, 1995a,b) that includes positive, negative, cognitive/disorganized, excitement, and depression factors. All interviews were conducted by trained research staff that had established inter-rater reliability on these measures (i.e., intraclass correlations greater than 0.80) in previous studies.

### Analysis

All analyses were performed in SPSS version 20. Data were analyzed first by recoding timed out trials as 0.5 correct (so that subjects who preferred to guess rather than time out on a trial would not have an advantage). Next, contextual *facilitation* was calculated as the proportion correct in the helpful condition minus that in the no-context condition, using only the 0.05° size difference difficulty trials (since the helpful condition included only this difference magnitude). Then, the amount of contextual *impairment* was calculated as the proportion correct in the misleading condition minus the proportion correct in the no context condition (all difficulty levels). C*ontext sensitivity* was the critical metric and corresponded to the difference between facilitation and impairment (with higher difference scores indicating greater sensitivity). Planned *t*-tests determined differences between pairs of groups on the context sensitivity variables. Context sensitivity was examined at each time point for each group and this was compared between groups. The groups were also compared across time points with a 3 (group) × 2 (context) × 2 (time) analysis of variance. Because the levels of the context factor were facilitation and impairment, a main effect of context is equivalent to significant context sensitivity.

## Results

### Demographic data

Means and standard deviations for demographic variables can be found in Table 1. The groups did not differ in gender composition: chi square (2) = 2.69, *p* > 0.26. As expected, there was a significant difference in age [*F*_(2, 68)_ = 12.84, *p* < 0.001], with the FEP group being younger than either the SCZ or CON groups (Scheffe *p*s < 0.001), who did not differ in age from each other (*p* > 0.97). Also as expected, the groups differed on education level [*F*_(2, 68)_ = 4.30, *p* < 0.05], with the CON group having more years of education than the SCZ (*p* < 0.05) but not FEP (*p* > 0.58) group, and the two patient groups not differing from each other (*p* > 0.29). There were no group differences for mother's education level or father's education level (*p*s > 0.70).

### Time 1

**Figure 3:**
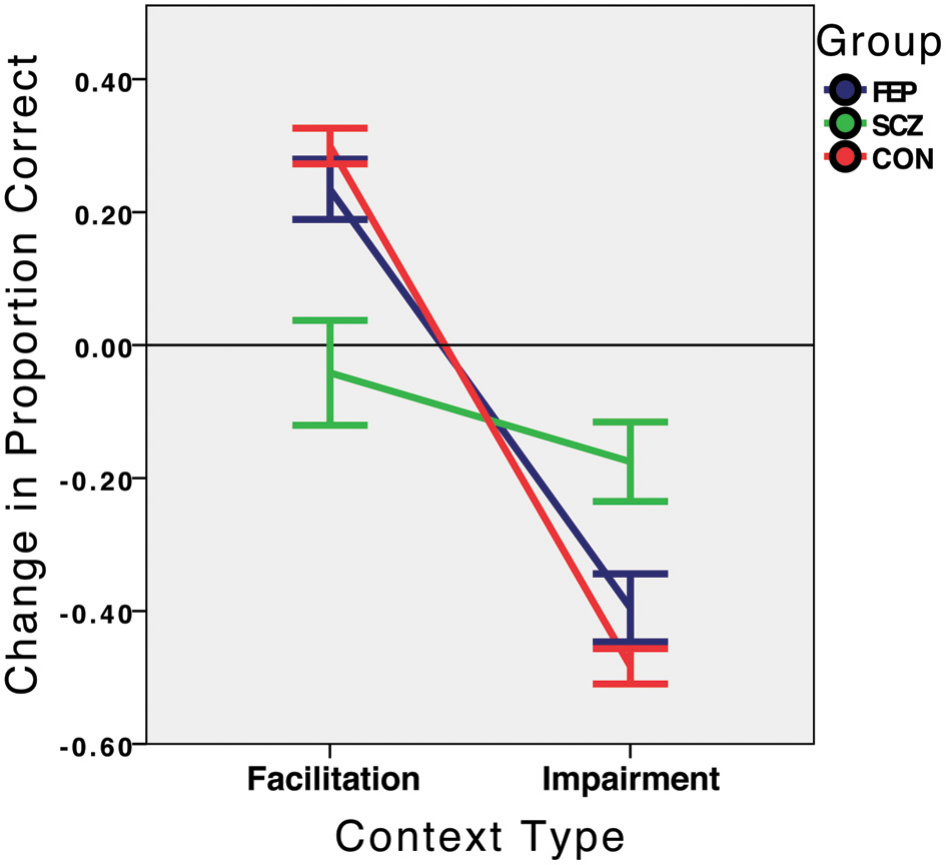
**Context sensitivity at Time 1 (hospital admission for the SCZ and FEP groups), by group**. Performance in the facilitation and impairment context conditions is expressed relative to performance in the no-context condition, which is represented by the value 0.0 on the *Y* axis. FEP, First Episode Psychosis; SCZ, Schizophrenia (multiple episode); CON, Healthy Control.

At initial testing (which for the patient groups, represented hospital admission), the CON group performed as expected (see Figure 3). The critical context sensitivity score (facilitation minus impairment) was enormous [*F*_(1, 26)_ = 397.46, *p* < 0.001, partial eta squared = 0.939], with strong facilitation [30.0%; *t*_(26)_ = 11.17, *p* < 0.001] and impairment [−48.3%; *t*_(26)_ = 18.16, *p* < 0.001]. Indeed, all 21 controls exhibited both facilitation and impairment. For the FEP group, there was also significant context sensitivity [*F*_(1, 15)_ = 65.01, *p* < 0.001, partial eta squared = 0.813], with significant contextual facilitation [23.4%; *t*_(15)_ = 5.18, *p* < 0.001] and impairment [−39.5%; *t*_(15)_ = 7.72, *p* < 0.001]. The SCZ group, however, exhibited no context sensitivity [*F*_(1, 20)_ = 1.14, *p* = 0.30), partial eta squared = 0.053], demonstrating a non-significant negative amount of facilitation [−4.2%; *t*_(20)_ = −0.53, *p* = 0.60] and some degree of impairment, although less than half as much as observed in the other groups [−17.5%; *t*_(20)_ = −2.94, *p* = 0.008]. Given the negative direction of the facilitation for the SCZ group, it is possible that some of the impairment arose simply because the surrounds had a general disruptive effect on performance regardless of the illusion, perhaps by making the target harder to isolate.

Context sensitivity was next directly compared between groups. The main effect of group on the context sensitivity metric was significant: *F*_(2, 61)_ = 17.03, *p* < 0.001, partial eta squared = 0.358. Planned comparisons indicated that the SCZ group was less context sensitive than the FEP [*t*_(32.1)_ = 3.32, *p* = 0.002] and CON [*t*_(23.6)_ = 4.90, *p* < 0.001] groups, whereas the FEP and CON groups differed only marginally from each other [*t*_(41)_ = 1.95, *p* = 0.059]. Of note, and as expected, the SCZ group was significantly *more* accurate than the FEP and CON groups in the misleading condition (69% vs. 52% vs. 46%; *p*s < 0.01 and 0.001, respectively), while being less accurate than these groups in the helpful condition (57% vs. 87% vs. 95%; *p*s < 0.005 and 0.001, respectively; see Table 2).

**Table 2:** **Percent correct in each condition in the Ebbinghaus illusion task, by group, at each time point**.

**Variable**	**TIME 1**						**TIME 2**					
	**SCZ (N = 21)**		**FEP (N = 16)**		**CON (N = 27)**		**SCZ (N = 14)**		**FEP (N = 14)**		**CON (N = 25)**	
	**M**	***SD***	**M**	***SD***	**M**	***SD***	**M**	***SD***	**M**	***SD***	**M**	***SD***
Helpful (0.05°)	56.8	41.3	86.9	23.6	94.5	7.4	77.2	7.4	81.4	23.8	90.9	20.5
Misleading (all difficulties)	68.5	23.4	51.8	18.3	45.9	13.9	58.0	13.9	51.2	17.0	51.1	15.0
No context (0.05°)	61.0	17.2	63.5	19.5	64.6	14.3	70.1	14.3	62.9	16.3	68.8	15.5
No context (all difficulties)	86.0	10.3	91.4	5.9	94.2	2.9	91.2	2.9	87.1	12.6	93.3	4.3

Next, correlations between context sensitivity and PANSS symptom factors were examined for each patient group separately. Despite the modest samples sizes, an interesting pattern of findings was revealed. Positive, depression and excitement symptoms positively correlated with context sensitivity in the SCZ group (*rho* = 0.45, *p* < 0.05; *rho* = 0.62, *p* < 0.005, and *rho* = 0.49, *p* < 0.05, respectively). In addition there were trends toward significant correlations with negative and cognitive/disorganized symptoms (*rho* = 0.40, *p* < 0.08; *rho* = −0.37, *p* < 0.10, respectively). Keeping in mind that the results are not corrected for multiple comparisons, these data suggest that poorer context sensitivity was associated with lower levels of positive, depression, excitement, and negative symptoms, and higher levels of disorganized symptoms. Similar to a prior study (Uhlhaas et al., 2006a), we dichotomized the conceptual disorganization score (P2 on the PANSS) so that SCZ subjects who had moderate or severe disorganization (>3; *n* = 7) were compared with those who had lower scores (*n* = 14). Replicating the past effect, we found that disorganized SCZ patients had significantly less context sensitivity [*t*_(19)_ = 2.24, *p* < 0.05]. This analysis could not be performed for the FEP group as only 2 FEP patients met criteria for the disorganized group. There were no significant symptom correlates for the FEP group (all *p*s > 0.55). These results are especially interesting because the symptom profiles of the FEP and SCZ groups did not differ on positive, negative, excitement, cognitive, or depression symptoms (*p*s > 0.12). Therefore, symptoms *per se* do not yield lower context sensitivity; *symptoms along with a more advanced illness do*.

It must be noted that the effect for positive symptoms was not expected. The more positive symptoms that a SCZ patient divulged during the PANSS interview, the more normal their perceptual performance (and hence more context sensitivity). This paradoxical inverse relationship in surround suppression tasks is not unprecedented (Yang et al., 2013) and will be discussed further below. Importantly, however, this symptom effect was driven primarily by depression. When both depression and positive symptoms were entered together as predictors of the context sensitivity index for SCZ patients in a multiple regression analysis (for which *R*^2^ = 0.44), depression (*B*eta = 0.56, *p* = 0.01) but not positive symptoms (*B*eta = 0.19, *p* = 0.36) was a significant predictor.

Finally, for the patient sample as a whole, poorer visual context sensitivity was significantly related to fewer depression (*rho* = 0.40, *p* = 0.01) and excitement (*rho* = 0.36, *p* = 0.03) symptoms, and—as expected—greater cognitive/disorganized symptoms (*rho* = −0.39, *p* = 0.019). When the patient sample as a whole was divided into those with and without conceptual disorganization, the group difference was significant: *t*_(34)_ = 2.31, *p* < 0.05.

### Time 2

At the second testing point (which, for the patient groups, represented hospital discharge or transfer), the sample sizes for each group were FE*P* = 14, SCZ = 14, and CON = 25. The smaller sample sizes for each group at discharge compared to admission reflected sudden discharges or transfers of patients that occurred before discharge testing could take place, or—in the case of 2 control subjects—unwillingness to return for a second session. There was a trend toward a group difference in subject attrition rates from Time 1 to Time 2: chi squared (2) = 5.91, *p* = 0.052. Importantly, in the SCZ group, which had the largest number of subjects unavailable to the study at Time 2, there were significant Time 1 differences between those whom could be tested at Time 2 (*n* = 14) versus those whom were unavailable (*n* = 7). The SCZ subgroup that was unavailable at Time 2 demonstrated, at Time 1, less context sensitivity [*F*_(1, 19)_ = 5.59, *p* = 0.029, partial eta squared = 0.237] than the SCZ subgroup that attended both sessions. Therefore, the between group data from Time 2 reported below are biased in a *conservative* direction by the loss of those patients with the least context sensitivity at Time 1, in the sense of our data potentially underestimating the magnitude of change in the SCZ group from Time 1 to Time 2. This is because poorer context sensitivity at Time 1 was (non-significantly) associated with *greater* improvement in context sensitivity from Time 1 to Time 2 for the SCZ group (*rho* = −0.35, *p* < 0.23). Moreover, although both the FEP and CON groups both demonstrated significant context sensitivity at Time 1, for both of these groups, poorer context sensitivity at Time 1 was also associated with greater improvement in context sensitivity across time points, with correlation values very similar to those observed in the SCZ group: FEP *rho* = −0.35, *p* = 0.23; CON *rho* = −0.36, *p* = 0.08. For both patient groups combined *rho* = −0.36, *p* < 0.07 and for the sample as a whole *rho* = −0.37, *p* = 0.007. Although only the correlation for the sample as a whole reached statistical significance, the values are remarkably similar across groups, and are all in the negative direction and indicative of a medium effect size (Cohen, 1992). All of this suggests that it is *un*likely that patients with the weakest context sensitivity at Time 1 would have had the smallest amount of improvement across Times 1 and 2, and thus that it is unlikely that the available data on change in performance over time for the SCZ group overestimate the true effect. It is worth noting as well that if the seven unavailable (at Time 2) SCZ patients, and the two unavailable subjects in each of the other two groups, were removed from the Time 1 analyses reported above, the context sensitivity, facilitation, and impairment differences between the SCZ and other groups would all remain significant (all *p*s < 0.005).

**Figure 4:**
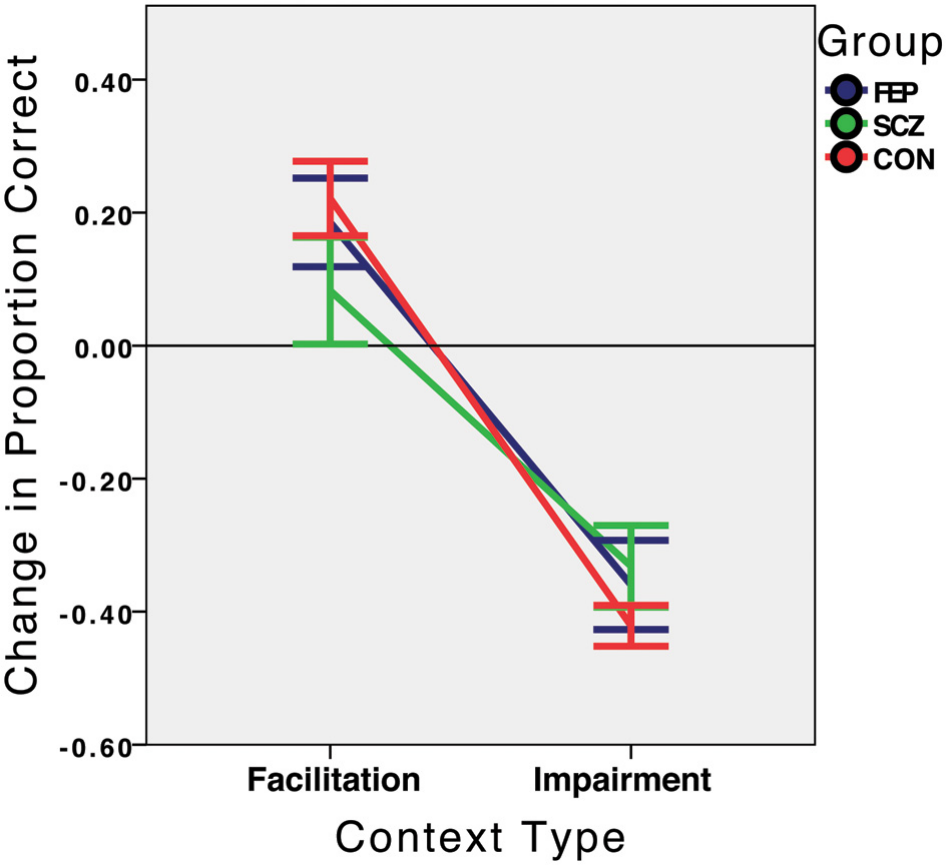
**Context sensitivity at Time 2 (hospital discharge for the SCZ and FEP groups, approximately 2 weeks after Time 1), by group**. Performance in the facilitation and impairment context conditions is expressed relative to performance in the no-context condition, which is represented by the value 0.0 on the Y axis. FEP, First Episode Psychosis; SCZ, Schizophrenia (multiple episode); CON, Healthy Control.

At discharge, as before, the CON group was strongly context sensitive [*F*_(1, 24)_ = 66.08, *p* < 0.001], partial eta squared = 0.734 (see Figure 4), with significant facilitation [22.13%; *t*_(24)_ = 3.95, *p* = 0.001] and impairment [−42.13%; *t*_(24)_ = −13.72, *p* < 0.001]. A similar, but somewhat weaker, pattern of performance was observed among FEP patients, who displayed context sensitivity [*F*_(1, 13)_ = 23.23, *p* < 0.001, partial eta squared = 0.641], facilitation [18.5%; *t*_(13)_ = 2.78, *p* = 0.016], and impairment (−36.0%; *t*_(13)_ = 5.37, *p* < 0.001). The SCZ group showed an entirely different pattern of behavior from hospital admission and a more similar pattern to those of the other groups, with significant context sensitivity [*F*_(1, 13)_ = 8.29, *p* = 0.013, partial eta squared = 0.389], significant impairment [−33.2%; *t*_(13)_ = −5.4, *p* < 0.001], and a non-significant, but positive, degree of facilitation [7.1%, *t*_(13)_ = 0.812, *p* = 0.432] in contrast to Time 1, when it was negative.

When contextual sensitivity was compared between groups, the overall effect of group was no longer significant (see Figure 4): *F*_(2, 50)_ = 0.513, *p* = 0.60, partial eta squared = 0.02), and none of the pairwise between-group comparisons approached significance (all *p*s > 0.12). There were no statistically significant correlations at Time 2 between PANSS symptom factor scores and context sensitivity for either the SCZ (all *p*s > 0.28) or FEP (all *p*s > 0.54) groups. Finally, for the patient group as a whole, poorer context sensitivity was marginally related to a higher level of cognitive/disorganized symptoms: *rho* = −0.32, *p* = 0.10, but there were no other symptom correlates (*p*s > 0.25). The comparison between disorganized and non-disorganized patients conducted at Time 1 was not conducted at Time 2 because only 3 patients had PANSS Conceptual Disorganization item scores greater than 3.

### Comparison between time 1 and time 2

We next conducted a 2 (context) × 2 (time point) × 3 (group) ANOVA, to examine whether group differences in context sensitivity became smaller over time. There was a main effect of context [*F*_(1, 50)_ = 124.62, *p* < 0.001, partial eta squared = 0.714], and a significant group × context interaction [F_(2, 50)_ = 4.38, *p* = 0.018, partial eta squared = 0.149] but no other effects (*p*s > 0.17), including the group × time × context interaction (*p* = 0.19). The significant two-way interaction of group × context is interesting because it shows that even with the loss of context-insensitive SCZ patients who were present at Time 1, and even when the data are collapsed across the two time points, the group difference on context sensitivity remains. To probe more sensitively for longitudinal group differences, we compared only the two extreme groups, CON and SCZ. Here, the group × context × time interaction depended marginally on the time point [F_(1, 37)_ = 2.90, *p* = 0.097, partial eta squared = 0.073], indicating that—across time points—the schizophrenia group became more like controls.

Next, we considered changes in symptoms and how those changes related to context sensitivity. We first note that there was a significant drop in all 5 PANSS factor scores from admission to discharge (all *p*s < 0.05) for both patient groups, with the exception of Excitement among the SCZ group, which remained stable over the 2 weeks in the hospital. This provides evidence that the PANSS scores were providing an accurate index of illness state and were sensitive to treatment effects in this study. More relevantly, changes in the PANSS scores and changes in context sensitivity were uncorrelated for the SCZ group (all *p*s > 0.16). For the FEP group, context sensitivity changes were positively correlated with changes in negative symptoms (*rho* = 0.56, *p* = 0.048) but not with other symptoms (*p*s >43). When all patients were combined, there were no statistically significant correlations between changes in symptoms and context sensitivity (*p*s > 0.51). We also considered whether context sensitivity at Time 1 could predict symptom changes across time points. It was found that—at Time 1—higher context sensitivity in the SCZ group predicted a greater reduction in positive symptoms from admission to discharge (*rho* = 0.58, *p* = 0.03), but not other symptom types (*p*s > 0.29). An important caveat is that none of the symptom correlates described in this paragraph were specifically predicted, and none would remain significant if corrected for multiple comparisons. Of note, the smaller sample sizes at Time 2 limit the ability to detect statistical significance. Thus, it will be interesting to observe whether two notable *rho* values for the SCZ group–between increases in context sensitivity over time and reductions in positive (−0.39) and excitement (−0.36) symptoms–hold up with continued data collection (see below).

## Discussion

Extending past findings, we found that—compared to healthy controls and people with a first episode of psychosis—persons with schizophrenia exhibit markedly less size contrast sensitivity at hospital admission (Uhlhaas et al., 2006a,b; Tibber et al., 2013). Consistent with other perception studies, (Silverstein et al., 1996; Uhlhaas et al., 2005; Silverstein and Keane, 2009; Keane et al., 2013), we also uncovered a state effect wherein the three groups demonstrated comparable context sensitivity by hospital discharge. Finally, we found that lessened context sensitivity may arise to some extent by the first episode of psychosis. It remains to be seen whether this is due to abnormal scores among a subgroup of FEP patients who go on to have schizophrenia as opposed to an affective psychosis. This question will be addressed in a subsequent report after longitudinal data are collected on this study sample.

A potential confound in the Time 1 findings is that SCZ patients may have demonstrated little context sensitivity not because of visual deficits but simply because they were not engaging in the task. It is possible that their advanced illness state caused them to randomly guess more often or occasionally press the wrong keys, leading to higher accuracy compared to other groups in the misleading condition and lower accuracy in the helpful condition. This explanation, if true, implies that the SCZ group should have performed worse than the FEP and CON groups in the no-context condition. It was found that the SCZ group's accuracy (86%) was about the *same* as that of the FEP group (91.4%, *p* = 0.17) but lower than that of the CON group (94.2%, *p* = 0.003). The direction of the difference is consistent with a generalized deficit, but the magnitude is not: a significant 8.2% dip in overall baseline accuracy doubtfully can explain a dramatic 64.9% group difference in context sensitivity (see Figure 3). Moreover, increased guessing among schizophrenia patients cannot explain why they performed far above chance in the misleading condition (69%) whereas controls and FEs performed right around chance (46% and 52%, respectively).

Another possibility is that SCZ patients became confused on the task precisely when a context was presented along with the targets: in these trials, subjects may have inadvertently judged the sizes of the surround circles rather than the central circles. However, if this were true, SCZ patients would have performed significantly better on the misleading than the helpful context trials, which did not occur (see Table 2). These results, taken together, indicate that there is a legitimate reduction in context sensitivity among people with schizophrenia.

### Symptom correlates

A paradoxical finding was that, in the SCZ group, more severe positive, excitement, negative and depression symptoms were associated with higher—and hence more normal—context sensitivity. Yang et al. (2013) found a similar effect with orientation and motion suppression tasks in which a central target is perceived to be more oriented or moving more in one direction when the surround contains elements are moving or oriented in the opposite direction. In that study, more pronounced Brief Psychiatric Rating Scale (BPRS) positive and negative symptom scores correlated with a greater effect of the surround (Yang et al., 2013). The effect sizes were not small (*r* = 0.67, *p* < 0.001 for motion; *r* = 0.49, *p* = 0.01 for orientation) and so cannot be dismissed as Type I errors. Why would more symptomatic patients behave more like healthy controls? Our finding that more normal context sensitivity is associated with increased positive, excitement, and depression symptoms may reflect both the typical co-occurrence of these symptoms, as well as their associated cognitive features. For example, positive symptoms and depression (which often includes agitation and excitement) often co-occur (Lindenmayer et al., 1991), and are related to a recent relapse as opposed to a chronically disabled state (Mulholland and Cooper, 2000; Hartley et al., 2013)—in other words, they are associated with patients who are higher functioning at their baseline. In terms of cognitive style, schizophrenia patients with more positive symptoms may allocate greater attentional resources to processing of the (irrelevant) contextual surrounds, leading to greater context sensitivity compared to the other groups. This is consistent with evidence of greater attentiveness to irrelevant cues being significantly correlated with positive symptoms (Morris et al., 2013). Yet another possibility is that some subjects, to varying degrees, are either unwilling or unable to be forthright about the true level of their symptoms. These subjects may have higher levels of impairment, poorer insight and prognosis, and more impaired visual processing. All of these explanations are speculative and, at this point, these unpredicted symptom correlates remain in need of further investigation.

Two symptom correlates that were predicted in our study were those between context sensitivity and cognitive disorganization in general and conceptual disorganization in particular. These effects replicate three earlier studies (Uhlhaas et al., 2006a,b; Horton and Silverstein, 2011), but not two recent ones (Tibber et al., 2013; Yang et al., 2013), which found no effect. Note however, that the Yang et al. (2013) study did not find evidence of a reduced Ebbinghaus illusion in schizophrenia, and studied clinically stable patients with little or no disorganization. A further difference between the Yang et al. and other studies, as noted by Tibber et al. (2013), is that the former study used unlimited stimulus presentation times, and this may diminish the illusion effect among all subjects, making significant correlations harder to detect. In addition, the task used by Yang et al. required comparing the size of a single circle to that of a circle with surrounds, and this method has been found to produce an illusion that is only about half as strong as when the sizes of two circles, each with surrounds, are compared (Franz et al., 2000), as was the case in the present study and other studies that found a reduced illusion effect in schizophrenia. The differences in patient samples between our study and the one by Tibber et al. (2013) may also explain why we found correlations between poorer context sensitivity and disorganization and they did not. Specifically, the patient sample in Tibber et al. (2013) was 79% outpatient, and 50% paranoid subtype, and only 3 of 24 patients scored greater than 3 on the PANSS Conceptual Disorganization item. This was, in general, a higher functioning sample than the one we studied (which was 100% inpatient), and the ones used in past studies where reduced context sensitivity and links to disorganization have been observed. In addition, paranoid subtype patients are generally higher functioning than disorganized patients and only typically begin to demonstrate disorganization after years of clinical deterioration, during which paranoid symptoms are reduced (McGlashan and Fenton, 1993). Moreover, disorganized symptoms are typically related to a poorer prognosis (Salokangas et al., 2002), and we have previously observed that reduced context sensitivity on an Ebbinghaus illusion task was more common in long-stay state hospital patients than in short-stay community hospital patients with schizophrenia (Uhlhaas et al., 2006b). In this study, positive and cognitive/disorganized symptom levels were independent of each other (*r* = −0.05, *p* = 0.83 at Time 1, and *r* = 0.07, *p* = 0.81 at Time 2), which was expected on the basis of prior factor analytic work (Lindenmayer et al., 1994a,b, 1995a,b) and the different relationships of these two factors with prognosis (Salokangas et al., 2002; Schennach-Wolff et al., 2011).

### Limitations

The data reported here have at least two important limitations. One is that the sample sizes are relatively small for the second time point, especially for the patient groups. Because the data reported here are preliminary findings from an ongoing study, later evidence will establish whether these findings are robust. The second limitation is that 1/3 of the SCZ patients who enrolled in the study were unavailable for testing at Time 2, as noted above (Results). This may have reduced the observed degree of change in context sensitivity in the SCZ group across time points. This suggestion is based on the findings that the SCZ patient subgroup that was unavailable at Time 2 was the least context sensitive at Time 1, and because patients (and controls) with the least context sensitivity at Time 1 demonstrated the greatest degree of change (improvement) across time points. It should be noted, however, that if our reasoning is incorrect and if those 7 SCZ patients' impairment at Time 1 was so severe that they would have *not* improved from hospital admission to discharge, then our available results would be biased in the direction of overestimating normalization of task performance in the SCZ group. Regardless, the available data make it clear that at least for the majority of SCZ patients tested (14/21 in this case), a clear perceptual impairment that is present at hospital admission, and that can not be accounted for by a generalized deficit, is not present at hospital discharge.

## Conclusion

This paper is the first report from an ongoing longitudinal study that investigates whether perceptual measures can predict symptom severity and/or level of functioning across multiple time points. To our knowledge, this is only the second study to examine longitudinal and treatment-related change in visual perception in schizophrenia (after Uhlhaas et al., 2005) and it is the first study of any kind to examine surround suppression in persons with a first episode of psychosis. In this preliminary report, we show that context sensitivity declines during acute phases of the illness, and—among schizophrenia patients—normalizes with short-term inpatient treatment. We also show that persons with first-episode psychosis exhibit marginally reduced context sensitivity at inpatient admission. These data suggest that performance on this Ebbinghaus illusion task may serve as a biomarker of relapse and recovery in people with schizophrenia. In later papers, we will report on how task performance, either at hospital discharge or at later time points, predicts short-term, post-hospital, prognosis and likelihood of relapse. We are especially interested in determining if first episode patients who demonstrate abnormal performance at either hospital-based testing have poorer outcomes, or if emergence of abnormal task performance over the next 1.25 years of illness is associated with a decline in functioning and/or a final diagnosis of schizophrenia versus a form of affective psychosis. Our other goal is to compare the predictive validity of perceptual task performance for established SCZ patients, relative to other putative biomarkers of treatment response for this population.

### Conflict of interest statement

The authors declare that the research was conducted in the absence of any commercial or financial relationships that could be construed as a potential conflict of interest.
